# Empowering communities, educating researchers: reflections on researcher training through the *‘Be the Voice of Your Community*’ Public and Patient Involvement (PPI) event

**DOI:** 10.1186/s40900-026-00957-7

**Published:** 2026-09-09

**Authors:** Caoimbhe Burke, Laura Fanneran, Prerna Shah, Elaine Quinn, Yvonne O’Meara, Amanda McCann, William M. Gallagher, Gayathri R. Devi, Anh N. Tran, Arman Rahman

**Affiliations:** 1 UCD School of Biomolecular and Biomedical Science, UCD Conway Institute, University College Dublin, Dublin 4, Dublin, Ireland; 2 Dublin, Ireland; 3 UCD Conway Institute of Biomolecular and Biomedical Research, University College Dublin, Belfield, Dublin 4, Dublin, Ireland; 4 Precision Oncology 2, Systems Biology Ireland, University College Dublin, Dublin 4, Dublin, Ireland; 5 Department of Surgery, Division of Surgical Sciences, Duke University School of Medicine, Durham, NC USA; 6 Department of Family Medicine and Community Health, Duke University School of Medicine, Durham, NC USA; 7 Cancer Risk, Detection and Interception, Duke Cancer Institute, Durham, NC USA; 8 UCD School of Medicine, University College Dublin, Dublin 4, Dublin, Ireland

## Abstract

**Background:**

Patient and Public Involvement (PPI) is increasingly recognised as essential in research design and funding. While benefits for patients include empowerment and trust in science, PPI also offers unique learning opportunities for early-stage researchers (ESRs), however, structured experiential PPI training for ESRs remains limited. The objective of this work is to report on the impact of a structured PPI engagement event, *Be the Voice in Your Community of Health Research* on ESRs’ confidence, skills, and understanding of PPI, while supporting patients from diverse ethnic backgrounds to share their lived experiences in cancer care and research.

**Methods:**

A one-day workshop held in 2025 at University College Dublin (UCD) combined short talks, patient panels and small-group discussions with facilitation. Each facilitation group included one senior researcher, who co-facilitated the session alongside the ESRs. The senior researcher provided oversight, supported discussion flow, and ensured methodological consistency, while the ESRs led participant engagement and interactive components. Participants comprised 5 senior researchers, 10 early-stage researchers (ESRs; PhD students and research assistants working in cancer biology, molecular oncology, bioinformatics and computational biology), and 20 community participants from minority ethnic backgrounds living in Ireland, including three individuals with lived experience of cancer. Post-event reflections and an anonymised questionnaire captured qualitative and quantitative feedback from the ESRs. Descriptive statistics summarised Likert-type items. Qualitative data were thematically analysed.

**Results:**

Nine of 10 ESRs rated the event *‘excellent’* (*one ‘good’*). Self-rated confidence in speaking with patients increased (mean of 4.3 before to 6.0 after; 7-point scale). Qualitative themes indicated (i) development of communication, (ii) active listening and empathy (iii) renewed research motivation and (iv) intention to adopt more inclusive research practices. ESRs showed a desire for more frequent training opportunities of this nature. Community participants reported feeling heard and respected and emphasised the importance of cultural awareness and interpersonal respect.

**Conclusions:**

This event demonstrated the dual value of PPI by creating a structured yet informal setting that fostered open dialogue and mutual learning, empowering patients while enriching researchers’ understanding and practice. Embedding structured PPI training early in research careers may help cultivate a generation of empathetic, socially responsible scientists and advance more inclusive, patient-centred cancer research.

**Supplementary Information:**

The online version contains supplementary material available at https://doi.org/10.1186/s40900-026-00957-7.

## Abstract (plain language summary)

This paper describes a hands-on training event that brought together early-stage cancer researchers (ESRs) and members of ethnic minority communities. Most researchers know patient and public involvement (PPI) matters, but do not receive practical training. At the ‘*Be the Voice of Your Community in Health Research’* event held in 2025, community participants shared real experiences of cancer care and research. Researchers practised listening and communication in small‑group discussions. Feedback showed that both researchers and patients found the experience valuable. Researchers felt more confident and reported a better understanding of patient perspectives while participants felt respected and heard. Face-to-face engagement helped researchers see how their work affects people in everyday life. Offering structured PPI training early in research can make involvement meaningful rather than tokenistic.

**Supplementary Information:**

The online version contains supplementary material available at https://doi.org/10.1186/s40900-026-00957-7.

## Introduction

### Background

PPI in research refers to active collaboration with patients, advocates and the public across, the research life cycle rather than treating them solely as study participants (Health Service Executive Research and [5]). There is growing evidence that integrating lived experience can significantly enhance research relevance, quality and impact. In Ireland, PPI is increasingly supported and expected by funders [3], aligning with the principle of working “*with* or *by* members of the public, rather than *to*, *about*, or *for* them” (Health Service Executive Research and [5]).

### The power of PPI

Meaningful PPI, i.e. collaboration between patients and researchers enhances the quality, relevance, and real-world impact of health research, but its success depends on deliberate planning, sustained support, and cultural change within research teams [7, 12], and has been shown to benefit services users and researchers alike [1]. In cancer research, PPI is particularly significant given the complex treatment journeys with varied social and cultural contexts. For example, PPI has revealed culturally mediated barriers to symptom recognition, help-seeking behaviour, and trial participation, which directly influence cancer outcomes but may be overlooked in standard research designs. Representation of ethnic communities is critical, as these groups often face barriers to both healthcare access and participation in research [9–11, 13]. A recent systematic review comprising of 12 qualitative and quantitative studies found that migrant populations and ethnic minorities, as well as individuals from lower socioeconomic backgrounds, are particularly vulnerable to health inequities and are less likely to participate in cancer screening programmes due to barriers including mistrust, limited culturally tailored communications, and structural inequalities [4]. These findings informed the rationale for our study by highlighting the importance of culturally sensitive engagement strategies and the need to involve underserved communities directly in dialogue about cancer research and healthcare access.

**Fig. 1:**
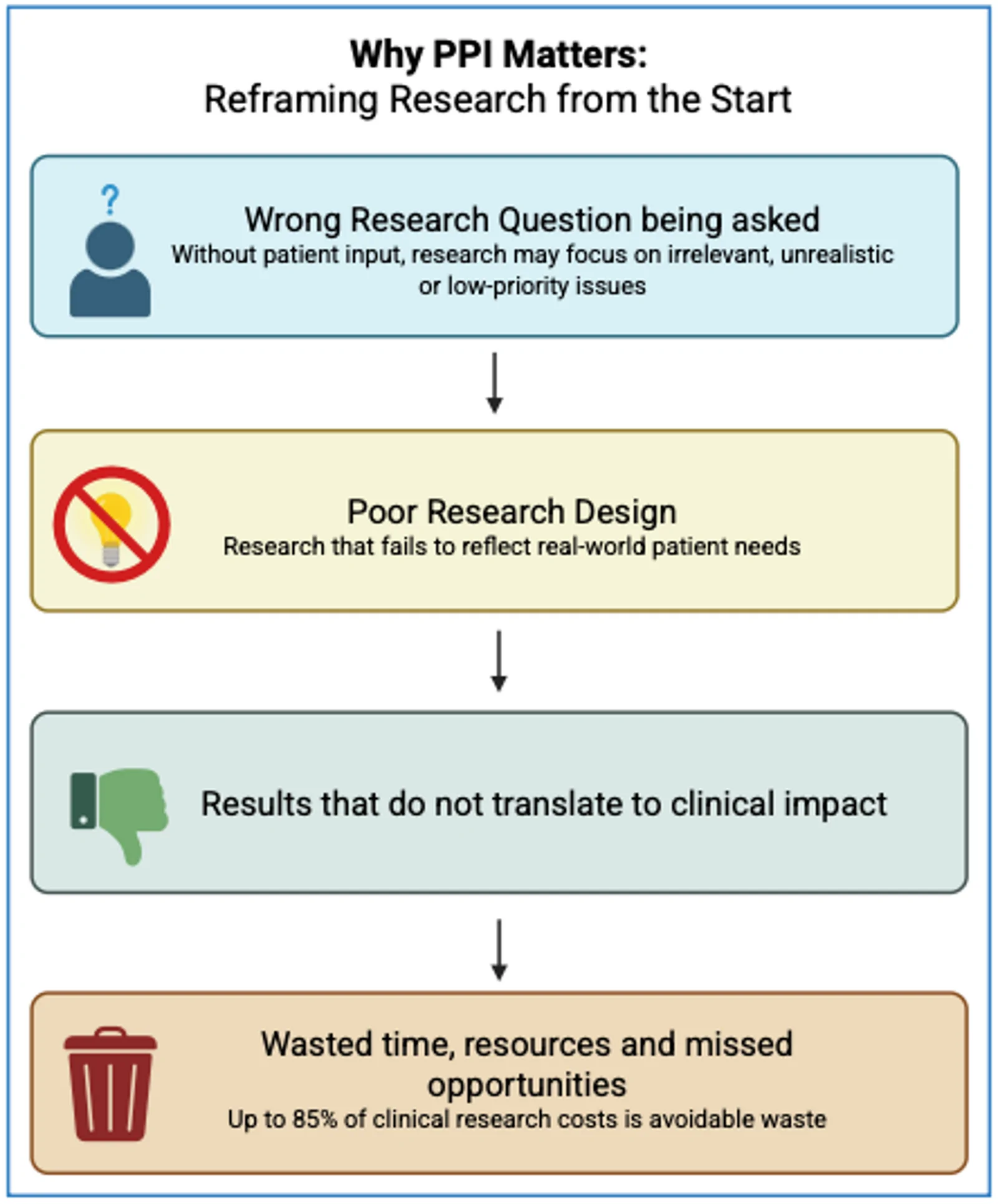
Why PPI matters: reframing research from the start [2]. Figure created using BioRender

For researchers, direct engagement reconnects scientific inquiry with real‑world need, helping prioritise questions likely to improve patient outcomes [14]. With an estimated 85% of clinical research considered wasted due to irrelevant or impractical research questions (Fig. 1) [2], early integration of patient voices can significantly improve research relevance, efficiency and ultimately impact. This approach reflects the principle: “*Nothing about us without us.”*

### Addressing the PPI training gap

Despite strong policy drivers, training for ESRs remains limited, leaving many ESRs uncertain about how to initiate respectful, effective involvement. Engaging in PPI requires more than just understanding its principles. It demands interpersonal skills, cultural sensitivity, and emotional intelligence. Without guidance and experiential learning, researchers may struggle to initiate conversations or build trust with patient collaborators, limiting the potential of PPI on their research. In turn, these issues risk rendering PPI efforts, superficial or tokenistic, potentially frustrating patients and undermining the very goals of involvement [6]. To harness the full value of PPI, it is therefore essential that researchers are comprehensively trained to engage with patients in ways that are fruitful with long term impact, for both the patient and researcher.

### The event and rationale

In response to this identified gap, the “*Be the Voice in Your Community in Health Research”* event was designed as a structured training day for ESRs in University College Dublin UCD, Ireland. Attendees included 10 ESRs (8 PhD students and 2 research assistants), five principal investigators/senior researchers serving as facilitators, patients, caregivers, and 20 ethnic minority community representatives, including three individuals with lived experience of cancer. The event aimed to create a safe, supportive space for researchers to learn how to meaningfully engage with patients from diverse ethnic backgrounds, while simultaneously providing a platform for patients to share their lived experiences of cancer care and research.

## Methods

### Event funding

A one‑day, workshop was designed to address this training gap and to promote bidirectional learning between ESRs and ethnic community participants. This event was supported by the UCD PPI Ignite Network and an Irish Cancer Society (ICS) Cancer Research Networking award (NA24RAH).

### Event design and timeline

The “*Be the Voice of your Community in Health Research”* workshop took place on the 12^th of^ February 2025, hosted at UCD Ardmore House, Belfield, Dublin. The programme combined short talks, a patient panel and four facilitated breakout discussions on six topics: (1) *Cancer awareness in ethnic minority communities*, (2) *Knowledge gaps in cancer research*, (3) *Access to cancer care and services*, (4) *Cultural sensitivity in health research*, (5) *Building trust in health research*, and (6) *Reflecting on the patient story: Empowering communities to get involved in health research activities (PPI)*. Group discussions in each breakout session were facilitated by a moderator and supported by a scribe, creating a space for open dialogue between patients and ESRs that encouraged mutual learning and the exchange of perspectives. To overcome language barriers that could affect both community participants and the ESRs during mutual dialogue, bilingual ESRs and community representatives were designated to provide translation and clarification support during the session.

The workshop opened with an introductory session, highlighting the significance of PPI in health research, and examples of international approaches to addressing cancer health disparities in underserved communities. This was followed by presentations showcasing creative science communication initiatives, linking patients and researchers, and discussions on how PPI can drive meaningful and immediate changes in cancer care and research. The morning concluded with a panel discussion, where patients shared their lived experiences within the Irish healthcare system, providing ESRs with first-hand insights into the challenges faced by individuals from diverse backgrounds.

After the morning break, structured breakout group discussions were conducted. In the breakout group discussions, ESRs acted as scribes, gathering and synthesising the key points and take-away thoughts that arose in their group discussion. This was followed by a reflective whole room discussion, where students became the spokesperson for their group to summarise and relay the points of their discussion to the wider audience. The event concluded with informal networking over lunch, where ESRs and patients could continue conversations in an unstructured environment, promoting free-flowing dialogue and relationship-building. ESRs were encouraged to summarise rather than speak on behalf of communities, and facilitators reminded groups to prioritise participants’ own words and perspectives.

### Participants and roles

Participants comprised three groups:

(i) **Community participants** (*n* = 20) included 3 individuals with lived experience of cancer and 17 members of the public from minority ethnic backgrounds living in Ireland, who identified personal or family migration backgrounds linked to six countries (India, Bangladesh, Venezuela, Bulgaria, Nigeria, and Iraq). This ensured that the event captured both lived-experience perspectives and broader public views while reflecting its multicultural context.
(ii) **ESRs** (*n* = 10), consisting of PhD students and research assistants from the UCD Conway Institute.
(iii) **Facilitators** (*n* = 5**)**, including principal investigators, senior researchers, and public engagement leads with prior PPI experience.

The scientific team for this study included senior researchers from Duke University, USA, and University College Dublin (UCD), Ireland, bringing together complementary expertise in cancer research and PPI. The author group also includes a patient research partner and community member, whose lived-experience perspective contributed to the development, evaluation, interpretation, and reporting of this work. As a co-author, they contributed to the drafting and revision of the manuscript, consistent with recognised good PPI practice. In addition to senior cancer researchers experienced in PPI, the team included a Psychosocial Oncologist and Systemic Psychotherapist, who provided support to patient representatives before, during, and after the event. Before the workshop, participants were offered preparatory conversations to discuss expectations and emotional readiness. During the event, the psychotherapist remained available for immediate emotional support and debriefing if sensitive issues arose. Following the workshop, participants were offered follow-up support and signposting to relevant psychosocial resources if required.

Consistent with participatory PPI principles, the programme was co-designed in close partnership with two Bangladeshi community leaders previously involved in the Invisible Spectrum initiative [10], who were recruited through established community networks and helped shape the aims, structure, and cultural framing of the event.

They met with the research team on three occasions over a three-month period to collaboratively shape the aims, structure, and cultural framing of the event. Their input informed participant engagement strategies, communication approaches, and delivery to ensure cultural appropriateness and community relevance, including the use of bilingual communication, respect for culturally sensitive topics, awareness of differing healthcare beliefs and norms, and facilitation styles that encouraged inclusive discussion.

Community partners played an integral role in the co-design and delivery of the initiative. While they were not involved in data analysis or manuscript development-primarily due to the defined scope of their involvement and practical considerations including timelines and language-they are acknowledged for their essential contributions in shaping the work.

Taken together, community members and researchers represented eleven different countries of origin (India, Bangladesh, Pakistan, Iran, Venezuela, Bulgaria, Italy, Ireland, Nigeria, Iraq, and Spain), underscoring the diversity and inclusivity of the workshop. Facilitators supported open dialogue and ensured psychological safety by establishing community agreements around confidentiality, respectful listening, optional participation, and the right to step out of discussions at any time.

The community participant contributions were central to the success of the event. The PPI panel was formed through an open call, inviting individuals to share their lived experiences, recognising the value of self-selection in empowering participants to tell their stories in a way that was meaningful to them. All expressions of interest were reviewed to ensure suitability and alignment with the aims of the workshop, with two applicants deemed not appropriate because their proposed contributions did not align with the cancer and health research focus of the event.

The three selected PPI panel members were supported and prepared in advance to share their experiences in a manner that respected their self-identified needs while also aligning with the workshop agenda. In recognition of their essential contribution, panel members were remunerated for their time and travel. By sharing personal stories, patients provided students with invaluable insights into the disease, which students may not have been exposed to before. Their stories highlighted everyday needs and problems that arise as a patient that can inform research strategies for ESRs to better address real-world issues. Additionally, these stories also highlighted challenges and systemic barriers for ethnic minority communities to contribute to and be part of meaningful PPI, an issue to be improved in the planning of any PPI event going forward.

The ESR role was to be actively involved in discussions, reflect on the patient experience and serve as scribes during breakout sessions. These activities were purposefully structured to strengthen their confidence and communication skills, by simultaneously navigating conversations with both patient representatives and academic professionals.

Finally, facilitators played a pivotal role in supporting both patients and students throughout the event. They guided discussions while ensuring that the sessions were inclusive and respectful by encouraging quieter participants to contribute, monitoring conversational balance, clarifying terminology when needed, and redirecting discussions if they became dismissive or exclusionary. All facilitators had prior experience with PPI-related work.

### Considerations and support

The event opened with community agreements covering respect, confidentiality and optional participation. Additionally, a designated support person was available throughout the day for anyone experiencing emotional distress. Participants with language barriers were supported throughout the sessions via translation provided by bilingual ESRs and, in one case, by a family member.

### Data collection and analysis

**Fig. 2:**
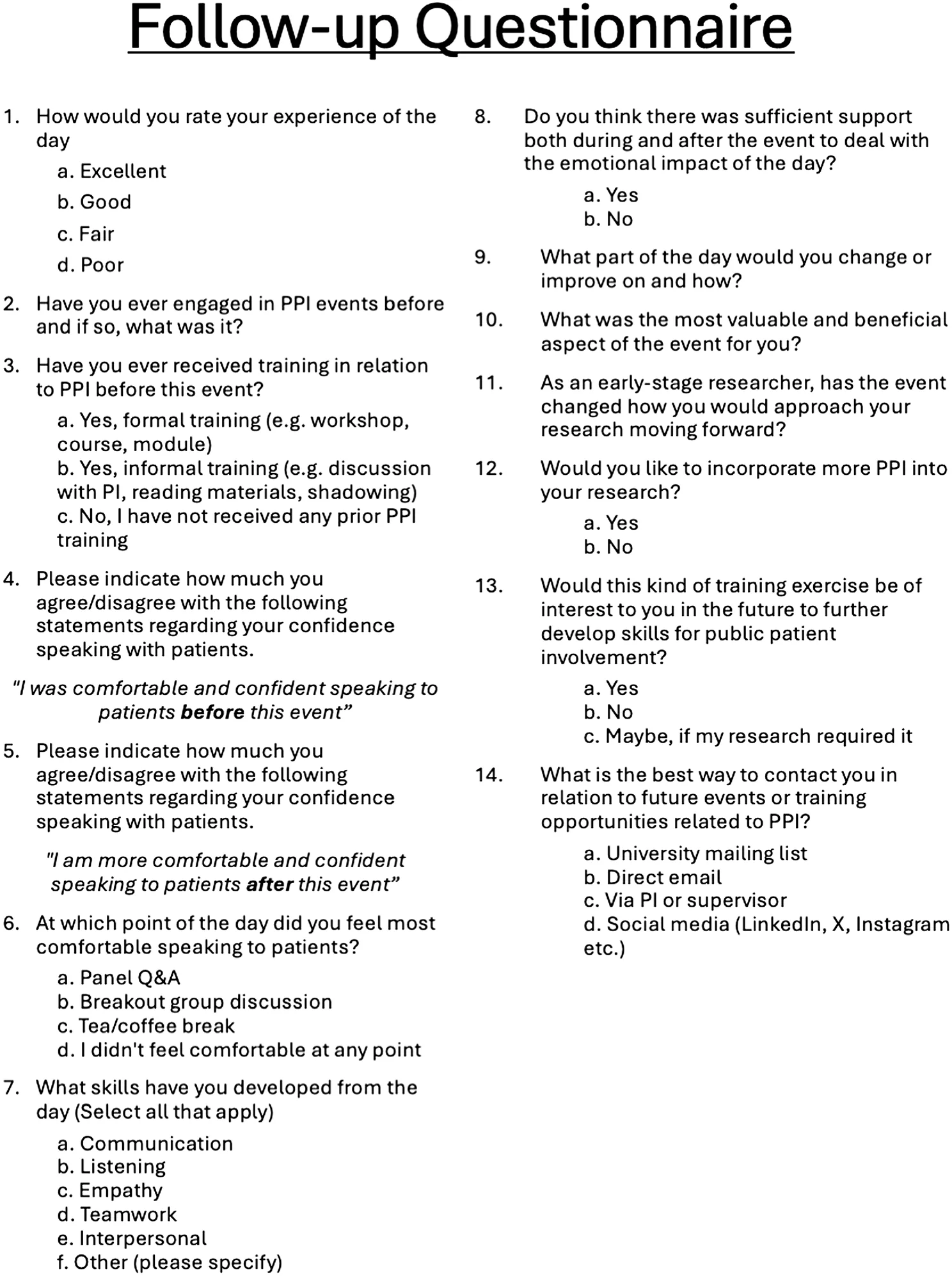
Questions covered by ESR follow-up questionnaire

To evaluate the impact of the training event, ESRs submitted brief written reflections (typically 100–250 words) responding to prompts such as: ‘What aspect of the event most influenced your understanding of PPI?’ and ‘Has this experience changed how you view your research or future engagement with patients/community members?’ ESRs also completed an anonymous feedback questionnaire containing multiple‑choice, Likert‑type and open‑ended items. Quantitative data were summarised descriptively. Qualitative data (written reflections and open-ended survey responses) were analysed using inductive thematic analysis, a bottom-up approach in which codes and themes were generated directly from the data rather than guided by pre-existing frameworks. This approach enabled the identification of recurring patterns related to PPI skills, attitudes, and intended changes to research practice (Fig. 2).

### Ethical considerations

This work constituted a service evaluation and researcher-training activity conducted in line with University College Dublin’s institutional policy on quality improvement and education initiatives. All participation was voluntary, and informed consent was obtained for involvement in discussions, and for the use of anonymised feedback in reporting. No identifiable personal information was collected or shared. Formal Research Ethics Committee (REC) approval was therefore not required.

## Results

The primary objective of the “*Be the Voice”* event was to strengthen ESRs’ skills, confidence, and understanding of meaningful PPI through structured dialogue with cancer patients from ethnic minority backgrounds. Through structured conversations and reflective exercises, students developed a deeper understanding of patient needs and the human context of their research. Findings are presented as a quantitative summary followed by three qualitative themes derived from post-event reflections and open-ended survey responses.

### Quantitative summary

Overall satisfaction was high. Specifically, 9 out of 10 ESRs rated the event *“excellent”* and 1 rated it *“good.”* Self-rated comfort speaking with patients increased from a mean of 4.3 pre-event to 6.0 post-event (7-point scale). “Comfort speaking with patients” was assessed using a self-reported Likert-type item capturing participants perceived confidence and ease in communicating with patients in research and PPI settings. Breakout discussions were consistently identified as the most valuable component. All ESRs (10/10) reported increased understanding of PPI, increased confidence engaging with patients, and a desire for further PPI training opportunities.

### Qualitative findings

Thematic analysis identified three overarching themes reflecting ESRs’ learning and development: (1) Growth in communication, active listening, and empathy; (2) Renewed motivation and reconnection to research purpose; and (3) Commitment to inclusive, community-engaged, and culturally sensitive research.

#### Theme 1: Growth in communication, active listening, and empathy

ESRs consistently reported that the small-group discussions created a safe environment to practice communicating with patients in a respectful, culturally sensitive way. Participants described becoming more aware of how tone, openness, and non-verbal communication influence trust. One ESR noted, *“It was clear that patients valued respect and cultural awareness far more than translation alone.”* Another added, *“Language can be translated*,* but true understanding happens through the kindness between words.”* Several ESRs commented that hearing views they had *“never even thought of before”* helped them recognise blind spots in their assumptions about patient needs. These exchanges supported the development of empathy, attentive listening, and confidence in engaging with diverse patient groups.

#### Theme 2: Renewed motivation and reconnection to research purpose

Direct engagement with community members reminded ESRs of the human impact behind their scientific work. Many reflected that the workshop *“refocused the priorities of my research*,*”* prompting them to look beyond data and reconnect with patient needs and lived experiences. One participant described feeling “*a burst of energy to push forward in my project*,” while another shared, *“It reminded me that my work isn’t just about generating data or publishing results but about making a real difference.”* These narratives highlight how experiential PPI can reorientate researchers toward purpose, relevance, and patient-centred inquiry early in their careers.

#### Theme 3: Commitment to inclusive, community-engaged, and culturally sensitive research

ESRs expressed a stronger commitment to embedding inclusive practices into their future research. Many highlighted learning about structural barriers such as waiting times, medical literacy challenges, and cultural norms that influence help-seeking and engagement. One ESR reflected that *“so much money and time and care go into research*,* and to see the benefits stifled by long wait times and cultural incompetence is disappointing.”* Another noted that the event *“reinforced my commitment to building respectful*,* long-term relationships with people from minority communities.”* ESRs also expressed interest in serving as cultural bridges, making research more accessible, and co-designing work that reflects real community priorities.

Together, these themes demonstrate how experiential PPI training can cultivate empathy, confidence, and social awareness among researchers. By embedding such experiences early in research careers, institutions can help develop a generation of scientists who approach their work with cultural sensitivity, compassion, and a commitment to inclusive, patient-centred research.

### Community participant feedback

Community participants (*n* = 20) reflected a range of cultural and migration backgrounds, with participants originating from six countries. The group included three individuals with lived experience of cancer, two of whom shared aspects of their personal cancer journeys during the panel discussion. Together, these contributions enriched the learning environment, while participants consistently reported feeling respected, welcomed, and genuinely listened to throughout the event.

They described the overall atmosphere as safe and comfortable, emphasising that the informal, conversational setup-small-group discussions with a facilitator and scribe-made it easier to speak openly about sensitive topics such as delays in diagnosis, cultural stigma, mistrust, communication barriers, and experiences of navigating the Irish healthcare system. Several participants contrasted this with traditional research events, noting that *“most events want us to fill in forms or answer questions*,* but here we could really talk.”*

Participants highlighted the value of being able to share experiences in their own words, noting that oral, group-based dialogue felt far more accessible than written surveys, particularly for those who face language barriers or who are more accustomed to storytelling and discussion as primary modes of communication. Individuals also appreciated the presence of facilitators who ensured respectful dialogue and explained concepts or terminology when needed, describing the day as *“a space where no one felt judged for asking questions.”*

Cultural sensitivity emerged as a core issue. Participants shared examples of how cultural misunderstandings or assumptions can affect healthcare interactions, such as difficulty advocating for oneself due to cultural norms around deference, limited familiarity with medical terminology, or fears of being perceived as disrespectful. One participant, discussing the financial burden of accessing primary care in Ireland, described having to **“*decide if this is €80 worth of pain*”** before seeking care, while another described travelling across multiple towns just to obtain a GP appointment. These accounts highlight systemic access barriers within primary care, particularly financial constraints and limited appointment availability that are experienced by wider segments of the population and not exclusively by ethnic minority communities. For individuals already facing social or cultural marginalisation, however, such barriers may be compounded, intensifying feelings of stigma and isolation and further influencing decisions to seek care. These discussions also highlighted how social and structural inequities may intersect with ethnicity, migration status, and healthcare access.

Cancer survivors in the group spoke about their diagnostic journeys, including lengthy delays, interpersonal experiences in clinical encounters, and the emotional toll of navigating unfamiliar systems. Their willingness to share these stories was viewed by other participants as empowering. Several noted that they learned from each other’s narratives as much as from the researchers. Some expressed gratitude that researchers were hearing experiences rarely captured in formal studies, stating that the day felt like *“a genuine partnership*,* not just research being done to us.”*

Finally, participants expressed enthusiasm for future involvement and emphasised the need for more events tailored to ethnic minority communities, particularly those who may not feel comfortable or represented in traditional research settings. Many shared that being invited into a dialogue, rather than positioned as research subjects, encouraged them to see how their perspectives could meaningfully influence cancer research and support broader community awareness.

## Discussion

An often-overlooked benefit of PPI is its positive effect on researchers’ motivation and sense of purpose. Participants in this study reported renewed energy and commitment to their work after engaging directly with patients. This aligns with Staley et al., [15], who found that researchers felt more resilient when their work clearly reflected patient priorities. Similarly, our participants described a strengthened belief in the relevance and credibility of their research through these interactions.

The impact of involvement in PPI is often examined from the perspective of patients or research outcomes, but rarely from that of the researchers themselves. This paper shifts the lens to focus on ESRs, revealing a clear demand for structured experiential PPI training. Despite many ESRs expressing a strong interest in PPI, few have the tools, experience and confidence to do so effectively. The “*Be the Voice”* event bridged that gap by creating a supportive environment in which researchers could practice active listening, empathetic communication and inclusive dialogue with patients and community partners. Our evaluation suggests that experiential, face‑to‑face PPI activities can quickly build ESR confidence and empathy, complementing policy guidance with practical experience. Consistent with prior work by Staley et al., [15], which demonstrates that direct contact with patients helps researchers “see the people behind the data,” our findings indicate that reluctance to implement PPI often reflects uncertainty rather than resistance [15]. While Ireland continues to expand formal PPI training opportunities, frameworks such as the UK Standards for Public Involvement developed by the National Institute for Health and Care Research (NIHR) may provide useful guidance for future development and evaluation of ESR-focused PPI training initiatives, particularly through their emphasis on inclusive opportunities, support and learning, working together, governance, communications, and impact [8].

Experiential and dialogue-based PPI training facilitates value-driven transformations that far exceeds what can be achieved through textbooks alone. In-person events allow for a deeper emotional and intellectual connection to the real-world implications of research, helping researchers become more socially attuned to the communities they aim to serve. Hence, PPI ensures that researchers are more socially conscious and attuned to the communities they aim to serve.

Dialogue-based models have reciprocal benefits: they strengthen researchers’ interpersonal skills while providing communities with accessible, respectful spaces for engagement [15]. Previous PPI events, including the *Invisible Spectrum* initiative-an annual public engagement programme developed by Precision Oncology Ireland (POI), part-funded by Science Foundation Ireland (under the Strategic Partnership Programme, Grant No. 18/SPP/3522) have shown that members of ethnic minority communities particularly value oral and inter-group communication for sharing and receiving information. *Invisible Spectrum* aims to promote cancer awareness, encourage screening uptake, and empower engagement with research among minority communities in Ireland, especially the Bangladeshi community, through bilingual discussions, presentations, and interactive activities [10]. The UCD-Duke collaboration in this publication further illustrates the feasibility of cross‑institutional partnership to co‑design practical PPI training and share perspectives on cancer health equity. However, we also recognise the importance of critically reflecting on representation and power. While ESRs occasionally summarised discussions during feedback sessions, facilitators emphasised that they should not speak over or replace community voices. Future initiatives should continue to explore co-facilitation and co-authorship approaches that minimise traditional researcher-participant hierarchies and avoid reproducing unequal power dynamics. Although this workshop was delivered on a relatively small scale, many elements of the format, such as facilitated discussions and community partnership approaches, may be adaptable to wider doctoral or ESR training programmes with appropriate institutional support and resources.

## Limitations

While this event provided valuable insights, several limitations should be acknowledged. This was a small, single‑institution activity assessed immediately post‑event using self‑report data. Detailed demographic data for community participants were limited, and longer‑term outcomes (e.g., sustained PPI practice, research design changes) were not measured. Future work should evaluate medium‑term impacts, include multiple institutions and disciplines, and incorporate richer demographic description.

While experiential learning offers valuable opportunities for researchers to develop empathy, communication skills, and confidence in engaging with diverse patient groups, it may have limitations when not supported by a clear theoretical framework or foundational skills in science communication. Experiential approaches alone may not fully equip ESRs to navigate complex communication challenges, such as addressing power dynamics, interpreting culturally shaped narratives, or translating patient insights into research practice. Prior literature highlights that without structured training, experiential engagement risks becoming informal, inconsistent, or overly dependent on individual interpersonal abilities, potentially limiting its effectiveness and sustainability [15]. Integrating experiential learning with formal instruction in communication theory and evidence-based best practices may therefore strengthen researcher preparedness and enhance the overall impact of PPI training.

## Conclusion

The ‘*Be the Voice’* event demonstrated clear dual value by both empowering communities and strengthening ESR’s competence for meaningful involvement in cancer research. It enhanced researchers’ understanding of patient perspectives, equipped them with practical tools to integrate PPI into their work, and highlighted strong demand for more frequent, formalised training of this kind. These findings suggest that embedding structured, experiential PPI education early in research careers-potentially within doctoral programmes-could normalise patient partnership as a core research competency and foster a more inclusive, patient-centred and socially responsible research culture.

## Supplementary Information

Below is the link to the electronic supplementary material.

Supplementary Material 1

## Data Availability

No datasets were generated or analysed during the current study.

## References

[1] Brett J, Staniszewska S, Mockford C, Herron-Marx S, Hughes J, Tysall C, Suleman R. A Systematic Review of the Impact of Patient and Public Involvement on Service Users, Researchers and Communities. Patient - Patient-Centered Outcomes Res. 2014;7(4):387–95. 10.1007/s40271-014-0065-0. 25034612

[2] Chalmers I, Glasziou P. Avoidable waste in the production and reporting of research evidence. Lancet. 2009;374(9683):86–9. 10.1016/S0140-6736(09)60329-9. 19525005

[3] Connor AE, Hughes C, Schäfer L, McNally L, Raw DO, Bahramian K, Carr B, Dunne IH, Lysaght J, Toole SAO, Simpson JC, Perry AS. Involving patients in healthcare research is well documented but can it work in lab-based research? Res Involv Engagem. 2023;9(1):90. 10.1186/s40900-023-00500-y. 37821914 PMC10568930

[4] De Marchi C, Di Lullo F, Ferrari C, Pettinicchio V, Sinopoli A, lombardo P, Tosti ME, Declich S, Pizzarelli S, D’Angelo F, Riccardi MT, Arrivi F, Forestiero FM, Rosca V, Romano A, Marotta D. Interventions to improve cancer screening adherence in migrants and ethnic minorities in the European Region: A systematic review. J Cancer Policy. 2026;47:100677. 10.1016/j.jcpo.2025.100677. 41390141

[5] Development HSERa. Guide to patient and public involvement in hse research. knowledge translation, dissemination and impact. A practical guide for researchers. 2024. HSE Retrieved 20/04/2025 from https://hseresearch.ie/wp-content/uploads/2021/12/Guide-no-8-Patient-and-Public-Involvement-in-HSE-Research.pdf.

[6] Gilfoyle M, MacFarlane A, Hannigan A, Niranjan V, Hughes Z, Salsberg J. The public and patient involvement imperative in Ireland: Building on policy drivers. Front Public Health. 2022;10:1038409. 10.3389/fpubh.2022.1038409. 36438293 PMC9684639

[7] Hewlett S, Wit Md, Richards P, Quest E, Hughes R, Heiberg T, Kirwan J. Patients and professionals as research partners: Challenges, practicalities, and benefits. Arthritis Care Res. 2006;55(4):676–80. 10.1002/art.22091. 16874772

[8] Moult A, Baker D, Aries A, Bailey P, Blackburn S, Kingstone T, Lwembe S, Paskins Z. Using the UK standards for public involvement to evaluate the public involvement sections of annual reports from NIHR managed research centres. Res Involv Engagem. 2023;9(1):109. 10.1186/s40900-023-00517-3. 38037160 PMC10688454

[9] Niksic M, Rachet B, Warburton FG, Forbes LJ. Ethnic differences in cancer symptom awareness and barriers to seeking medical help in England. Br J Cancer. 2016;115(1):136–44. 10.1038/bjc.2016.158. 27280638 PMC4931374

[10] O’Grady S, Ralston JC, McKiernan E, Lanigan F, Salauddin M, Sharmin S, McVeigh T, Gallagher WM, Guerin S, Rahman A, Kolch W. Invisible spectrum: a model for minority community public engagement in cancer research. medRxiv. 2024;20242012200224317617. 10.1101/2024.12.02.24317617. PMC12100985 40410864

[11] Redwood S, Gill PS. Under-representation of minority ethnic groups in research–call for action. Br J Gen Pract. 2013;63(612):342–3. 10.3399/bjgp13X668456. 23834862 PMC3693777

[12] Schilling I, Gerhardus A. Is this really Empowerment? Enhancing our understanding of empowerment in patient and public involvement within clinical research. BMC Med Res Methodol. 2024;24(1):205. 10.1186/s12874-024-02323-1. 39272031 PMC11395860

[13] Sonawane K, Alekseyenko A, Magwood GS, Mehrotra S, Devi GR, Ford ME. Trust in researchers and willingness to engage in cancer research. Oncologist. 2026;31(6). 10.1093/oncolo/oyag170. PMC13157339 42063223

[14] Staley KChanging what researchers’ think and do’: is this how involvement impacts on research? Res All. 2017;1(1):158–67. 10.18546/RFA.01.1.13.

[15] Staley K, Abbey-Vital I, Nolan C. The impact of involvement on researchers: a learning experience. Res Involv Engagem. 2017;3(1):20. 10.1186/s40900-017-0071-1. 29062545 PMC5611580

