# Supplementary material for: Empowering communities, educating researchers: reflections on researcher training through the ‘Be the Voice of Your Community’ Public and Patient Involvement (PPI) event
Source: Res Involv Engagem. 2026 Sep 9;12:142. doi: 10.1186/s40900-026-00957-7 (PMC13560106; doi:10.1186/s40900-026-00957-7)
Supplement: Supplementary file 1 [file 40900_2026_957_MOESM1_ESM.docx]

**Guidance for Reporting Involvement of Patients and the Public – Short Form**

**Manuscript Title:**
*Empowering Communities, Educating Researchers: Reflections on Researcher Training Through the ‘Be the Voice of Your Community’ Public and Patient Involvement (PPI) Event*

**1. Aim of Patient and Public Involvement (PPI)**

The aim of patient and public involvement (PPI) was to ensure that the design, delivery, and learning outcomes of the “Be the Voice in Your Community in Health Research” event were culturally appropriate, community-informed, and responsive to the needs of ethnic minority communities traditionally underrepresented in research engagement. The initiative sought to create a safe and inclusive environment where community members could share lived experiences of cancer care while early-stage researchers (ESRs) developed practical skills in communication, empathy, and meaningful involvement. PPI aimed to promote mutual learning, strengthen trust between researchers and communities, and support culturally sensitive approaches to cancer research engagement.

**2. Methods Used for PPI**

The programme was co-designed in partnership with two Bangladeshi community leaders who collaborated closely with the research team through three structured planning meetings conducted over a three-month period prior to the event. These meetings informed the event’s objectives, structure, discussion topics, communication approaches, and cultural framing to ensure accessibility and relevance for participants from diverse ethnic backgrounds.

Community partners advised on recruitment strategies, culturally appropriate language and facilitation styles, and approaches to supporting participants who might face language or cultural barriers. The event incorporated patient panels, facilitated small-group discussions, and informal networking opportunities designed to promote dialogue rather than one-directional information sharing. Bilingual participants supported communication where required.

Community participants, including individuals with lived experience of cancer, actively contributed during the event through storytelling, dialogue, and shared reflections. A Psychosocial Oncologist and Systemic Psychotherapist provided support before, during, and after the event to ensure psychological safety and participant well-being. Community agreements addressing respect, confidentiality, and voluntary participation were established at the outset.

**3. Results of PPI in the Study**

Community involvement directly shaped the structure and atmosphere of the event, resulting in culturally sensitive discussions and an environment that encouraged open dialogue about cancer experiences, healthcare access, and research participation. Community-informed recruitment and facilitation approaches enabled participation from individuals representing multiple cultural backgrounds and levels of research familiarity.

Community narratives provided ESRs with insights into lived experiences, cultural barriers, and systemic challenges not typically encountered in laboratory-based training. Feedback indicated that participants felt respected, listened to, and valued as contributors rather than research subjects. ESRs reported increased confidence communicating with patients, improved empathy and listening skills, renewed motivation for their research, and a stronger commitment to inclusive research practices.

**4. Discussion and Conclusions (Impact of PPI)**

PPI strengthened the initiative by ensuring that the training experience reflected community priorities and real-world perspectives. Collaboration with community leaders enhanced cultural relevance, trust-building, and inclusivity, enabling meaningful dialogue between researchers and participants. The event demonstrated that experiential, co-designed PPI activities can simultaneously empower community members and provide impactful training for researchers.

Findings suggest that early integration of structured PPI experiences within research training can foster culturally aware, patient-centred researchers and promote more socially responsive cancer research practices. The co-design approach contributed to the dual outcomes highlighted in the study: community empowerment and researcher development.

**5. Reflections/Critical Perspective**

Community partners played an integral role in co-designing and implementing the event; however, they were not involved in data analysis or manuscript development and therefore do not meet formal authorship criteria (e.g., contribution to analysis and manuscript drafting/approval). Their contributions are acknowledged within the manuscript.

The process highlighted several strengths, including trust-building through community partnership, the value of dialogue-based engagement, and the effectiveness of culturally responsive facilitation. Challenges included balancing academic timelines with community availability, managing linguistic diversity, and ensuring sustainable pathways for continued involvement beyond single events.

Future initiatives will aim to support deeper community involvement in dissemination activities and, where feasible, co-authorship and co-production of research outputs.

Clarification of community involvement in design and delivery
We have expanded the Methods section (page 7) to more clearly describe the extent of community involvement. Specifically, we now detail the co-design process with community leaders, including structured planning meetings over a three-month period, and how their input shaped the event’s aims, structure, cultural framing, and participant engagement strategies.

Explanation for absence of community co-authors
We have added a brief statement (page 7) clarifying the roles of community partners in co-design and implementation. We note that while their contributions were integral, they were not involved in data analysis or manuscript development and therefore do not meet authorship criteria. Their contributions are formally acknowledged.
